# A case of *Candida metapsilosis* conjunctivitis in a neonate admitted to the cardiac heart intensive care unit

**DOI:** 10.1002/ccr3.6870

**Published:** 2023-01-23

**Authors:** Shima Aboutalebian, Arezoo Charsizadeh, Hamid Eshaghi, Bahram Nikmaesh, Hossein Mirhendi

**Affiliations:** ^1^ Department of Parasitology and Mycology, School of Medicine Isfahan University of Medical Sciences Isfahan Iran; ^2^ Mycology Reference Laboratory, Research Core Facilities Laboratory Isfahan University of Medical Sciences Isfahan Iran; ^3^ Immunology, Asthma, and Allergy Research Institute Tehran University of Medical Sciences Tehran Iran; ^4^ Department of Infectious Disease Tehran University of Medical Sciences Tehran Iran; ^5^ Department of Medical Laboratory Science School of Allied Medical Science, Tehran University of Medical Science Tehran Iran; ^6^ Zoonoses Research Centre Tehran University of Medical Sciences Tehran Iran

**Keywords:** antifungal susceptibility testing, *Candida metapsilosis*, conjunctivitis, identification, pediatrics

## Abstract

Harmless commensal *Candida* species, especially uncommon and rare ones may rarely cause a serious infection. *Candida metapsilosis* is a recently described yeast that is phenotypically indistinguishable from *Candida parapsilosis* and molecular methods are essential for its identification. We report the first case of *Candida* conjunctivitis due to *C. metapsilosis* obtained from the eye discharge of a 40‐day‐old girl with congenital heart disease admitted to the cardiac intensive care unit (CICU). The yeast isolate was identified by sequencing the entire ITS1‐5.8 rRNA‐ITS2 region. Antifungal susceptibility testing performed according to the CLSI M27‐A3 showed that the isolate was susceptible to amphotericin B, fluconazole, itraconazole, voriconazole, clotrimazole, nystatin, terbinafine, 5‐fluorocytosine, and caspofungin. Differentiation of the fungal new species allows us the accurate diagnosis and treatment, and a better understanding the microbial epidemiology.

## 1 INTRODUCTION

Due to an increasing number of predisposed patients and developments in medical interventions with the increasing number of invasive medical procedures, some *Candida* species previously considered to be harmless commensals are emerging as causes of serious disease.^1^ Fungal ocular infections are extremely rare, but if not treated they can be very harmful and may lead to permanent vision loss. *Candida* species can cause conjunctivitis as a result of an eye injury or trauma which is subsequently transmitted to the bloodstream.^2^, ^3^ *Candida parapsilosis* sensu lato is one of the common fungi causing conjunctivitis.^1^ It is ubiquitous yeast in nature and found in many environments. It is the most frequently colonized species, isolated from the subungual space of human hands; therefore, can spread nosocomially through hand carriage. It has been frequently associated with infections in newborns.^1^

As a recently described *Candida* species*, C. metapsilosis* is phylogenetically closely related to *C. parapsilosis* complex but is vary in geographic and anatomic prevalence, as well as in antifungal resistance characteristics.^4^ Using genotypic methods, the clonally related species of *C. parapsilosis* complex were subsequently divided into three distinguished species: the more prevalent *C. parapsilosis* sensu stricto, and two newly designate species *C. orthopsilosis* and *C. metapsilosis*.^5^, ^6^ It has been reported that 1%–10% of the *C. parapsilosis* isolates identified through conventional biochemical tests are indeed *C. metapsilosis* or *C. orthopsilosis*.^7^

We present a case of *C. metapsilosis* infection associated with conjunctivitis in a 40‐day‐old girl who was undergoing heart surgery and was admitted to the cardiac intensive care unit. To the best of our knowledge, this is the first case of *C*. *metapsilosis* in Iran.

## 2 CASE REPORT

A 40‐day‐old girl candidate for heart surgery was admitted to Children's Medical Center in Tehran, Iran, after being diagnosed with congenital heart disease. She was treated with some related medicines and also sulfacetamide sodium ophthalmic solution to prevent/treatment of bacterial eye infections such as conjunctivitis. On the twelfth day of surgery and admission to CICU, she presented a discharge from the left eye. Physical examination showed eyelid swelling and erythematous conjunctiva with mucopurulent discharge. There was no evidence of obvious trauma, intraocular inflammation, or cornea involvement. The treatment included 5% Natamycin ophthalmic suspension every 6 h for 7 days. Microbiological investigations were performed by culturing purulent ocular discharge on blood agar, MacConkey agar, and Sabouraud dextrose agar which yielded yeast colonies in pure cultures and were reported as *Candida* spp. based on performing the Germ Tube test. The isolates were subsequently cultured on CHROMagar Candida medium for purification and primary identification and identified as *C. parapsilosis*. Also, the PCR‐restriction fragment length polymorphism (PCR‐RFLP) assay was described for rapid confirmation of identification.^8^

DNA of the yeast isolate was extracted using the boiling method; briefly, a few colonies of the overnight culture growth were transferred to a 1.5 ml tube containing 50 μl of sterile distilled water and placed in boiling water for 20 min, centrifuged for 10 min at 2000 *g*, and the supernatant used as the DNA template.^9^ The ITS1‐5.8 S‐ITS2 region was PCR‐amplified by using ITS1 and ITS2 primers as described previously,^10^ and the product was Sanger‐sequenced using the forward primer. Based on NCBI and ISHAM barcoding databases, the isolate was identified as *Candida metapsilosis*. The sequence was deposited in the GenBank under accession number OP961717. In vitro susceptibility of the isolate to amphotericin B, fluconazole, itraconazole, voriconazole, clotrimazole, nystatin, terbinafine, 5‐fluorocytosine, and caspofungin was tested according to M27‐A3 standard of the clinical and laboratory standards institute (CLSI),^11^ and the minimum inhibitory concentrations were 0.5, 2, 0.125, 0.015, 0.015, 1, 0.015, 0.063, and 0.125 μg/ml, respectively.

## 3 DISCUSSION

Fungi can be recovered from the normal conjunctival sac. Fungal conjunctivitis is usually secondary to inflammation of the cornea, lacrimal sac, and tear ducts, and depends on the causative agent manifests with different symptoms such as acute inflammation of the conjunctiva with mucopurulent symptoms^12^ as what was observed in our patient, and also as a salmon‐pink tumor in the bulbar conjunctiva,^13^ punctate epithelial keratoconjunctivitis,^14^ conjunctivitis with giant papillae,^15^ and conjunctival candidiasis mimicking ocular surface squamous neoplasia.^16^ *Candida* conjunctivitis has been described mainly in the newborns and school children,^2^ however, it is a rare disorder with low incidence, on the one hand probably due to the vascular network and lymphoid structures of the conjunctiva which provide abundant cellular defense, and on the other hand, because of difficulty in diagnosis due to the lack of specific features and clinical findings.^15^, ^17^ *C. parapsilosis* sensu *lato* and its cryptic species have become the predominant causes of candidemia in some pediatric settings, especially in newborns. They are responsible for 17%–50% of bloodstream fungal infections episodes.^18^ The incidence of fungemia caused by *C*. *orthopsilosis* and *C. metapsilosis* has been increasingly reported in recent years.^19^, ^20^, ^21^ *C*. *metapsilosis* is a rare entity in nosocomial *Candida* infections. Previous studies adjusted the frequency of infections attributed to *C*. *metapsilosis* from 0% to 35.5% of all *C*. *parapsilosis* sensu *lato* around the world.^22^ Studies suggested that *C. parapsilosis* sensu stricto and *C*. *orthopsilosis* are existed as human commensal, while *C. metapsilosis* is an environmental organism,^1^ accordingly, the high rate of isolation of *C. metapsilosis* in a local area of China may be attributed to exogenous origins.^23^

*Candida parapsilosis* sensu *lato* was isolated from soil, plants, domestic animals, insects, seawater, and marine environment, as well as from skin, gastrointestinal tract, and mucosal surfaces including the eye, vaginal, and birth canal.^1^ That is one of the main species of the microflora of the subungual space.^1^ As the conjunctival sac is generally sterile at birth, therefore, there are two manners for a neonate to develop *C. parapsilosis* conjunctivitis. One possible way is eye colonization in neonates via the birth canal and post‐antibiotic invasion after the use of antibacterial drops in neonate settings, hence colonization precedes infection, and another is the eye injury through contact with the colonized hands and fingernail beds of the nursing staff.^1^ Accordingly, conjunctivitis caused by *Candida* species in newborns may be a common occurrence.^2^, ^17^, ^24^ A case of horizontal transmission of *C*. *parapsilosis* transmitted from the hands of two nurses to the neonate's conjunctiva and then to the bloodstream was reported.^2^ Our case suggests that *C*. *metapsilosis* can also be a human commensal, although more researches are needed.

There are conflicting assessments in the context of virulence and antifungal susceptibility pattern of *C*. *metapsilosis*.^7^, ^18^, ^20^, ^25^, ^26^ This species has been reported as the least virulent member of the *C*. *parapsilosis* complex; the low frequency in clinical settings could be associated with this property. Cell cultures exposed to *C*. *metapsilosis* release the least hydrolytic enzymes and hemolytic factors, and represented the least biofilm production and pseudohyphae formation.^22^, ^25^ Nonetheless, recent data indicated an increasing role of *C*. *metapsilosis* in human mycoses, especially in blood‐stream infections, and its isolation from blood cultures deserves particular attention.^23^ It seems that a strain‐dependent virulence mechanism might contribute to the invasiveness of this non‐virulent yeast.^23^

It is remarked that accurate discrimination among cryptic species of *C. parapsilosis* seems unnecessary due to similar antifungal susceptibility profile to commonly used azoles and amphotericin B. However, concerning echinocandins, identification to the species level is necessary because of different susceptibility patterns.^23^ Although it is reported that *C*. *orthopsilosis* sensu stricto is less susceptible to amphotericin B, echinocandins, and fluconazole than *C*. *metapsilosis* and *C*. *orthopsilosis*, Canton et al. showed a high susceptibility of these two cryptic species to nine antifungal.^20^ Hence, accurate characterization and discrimination of these cryptic species are important in the two aspects of epidemiological surveys and antifungal susceptibility pattern. In any case, it should be remembered that fungal conjunctivitis causes serious complications and requires intensive antifungal therapy, therefore, early diagnosis and rapid antifungal treatment play a significant role in the course of the disease and reduce complications such as permanent vision loss.^12^

## 4 CONCLUSION

Infections by *C*. *metapsilosis* can be attributed to nosocomial transmission by hand carriage or vertical transmission. Differentiation and susceptibility testing of this new species allows for defining the epidemiology and targeted treatment. Accurate identification needs molecular methods such as ITS sequencing.

## AUTHOR CONTRIBUTIONS

**Shima Aboutalebian:** Conceptualization; data curation; formal analysis; investigation; methodology; resources; validation; visualization; writing – review and editing. **Arezoo charsizadeh:** Data curation; formal analysis; methodology; resources; visualization; writing – original draft; writing – review and editing. **Hamid Eshaghi:** Data curation; visualization; writing – review and editing. **Bahram Nikmaesh:** Data curation; visualization; writing – review and editing. **Hossein Mirhendi:** Funding acquisition; methodology; project administration; supervision; validation; visualization; writing – review and editing.

## FUNDING INFORMATION

This work was supported by Isfahan University of Medical Sciences, Isfahan, Iran (grant No. 1400180).

## CONFLICT OF INTEREST

None.

## CONSENT

Written informed consent was obtained from the next of kin of the patient for the publication of any data included in this article.

## Data Availability

The data that support the findings of this study are openly available in NCBI at http://www.ncbi.nlm.nih.gov/blast.
